# The current status of “spirituality and health” teaching in Brazilian medical schools: a nationwide survey

**DOI:** 10.1186/s12909-023-04153-z

**Published:** 2023-03-20

**Authors:** Giancarlo Lucchetti, Paulo Othavio de Araujo Almeida, Elena Zuliani Martin, Leonardo Garcia Góes, Keylla Cássia Gomes Cotta, Andressa Correia Lima, Oscarina da Silva Ezequiel, Alessandra Lamas Granero Lucchetti

**Affiliations:** 1 School of Medicine, Federal University of Juiz de Fora, Juiz de Fora, Brazil; 2 School of Medicine, Federal University of Mato Grosso, Mato Grosso, Brazil; 3 School of Medicine, Centro Universitário São Camilo, São Camilo, Brazil; 4 Escola Superior de Ciências da Saúde, Porto Alegre, Brazil; 5 School of Medicine, Universidade Federal de Juiz de Fora, Dom Bosco – CEP, Av. Eugênio do Nascimento s/n° Bairro, 36038-330 Juiz de Fora, MG Brazil

**Keywords:** Medical education, Spirituality, Religion, Teaching, Medical students

## Abstract

**Background:**

Recent data on the teaching of “spirituality and health” (S/H) in medical schools are needed. In this study, we aimed to investigate the current status of S/H teaching in Brazilian medical schools, the opinions of medical directors/deans on this topic and the factors associated with its incorporation into the curriculum.

**Methods:**

A nationwide cross-sectional survey was carried out in 2021. Information concerning the S/H content in the curricula of medical schools was obtained through medical school representatives and other sources. Medical school representatives were asked about their opinions of and barriers to S/H teaching. Regression models were used to evaluate the factors associated with the incorporation of such content into the curriculum.

**Results:**

Information on the incorporation of S/H content in medical curricula was retrieved from different sources for all 342 (100%) Brazilian medical schools. Among the representatives, 150 (43.9%) completed the online form. An increase in the S/H content in Brazilian medical schools was observed (from 40% to 2011 to 65.5% in 2021). Most medical school representatives agreed that this issue is important in medical training and that more space in the curriculum is needed. However, they also observed several barriers, such as a lack of knowledge of medical teachers/faculty, a lack of time, and the topic not being included in teaching plans. The most important factors that influenced the incorporation of S/H teaching in medical schools and representatives’ opinions were a lack of time and knowledge, professor preparedness and standardized national competency requirements.

**Conclusion:**

These results could help medical educators rethink the incorporation of S/H content into their curricula.

**Supplementary Information:**

The online version contains supplementary material available at 10.1186/s12909-023-04153-z.

## Introduction

The field of “Spirituality and Health” (S/H) has been constantly growing in the scientific community, stimulated by advances in research and by the recommendations of medical organizations such as the American College of Physicians, the American Medical Association and the World Health Organization [1]. Growing evidence has motivated medical schools to include S/H teaching in their curricula, aimed at increasing patient-centered approaches and fostering holistic and inclusive medicine [2–4].

In the last few decades, medical schools all over the world have incorporated S/H content into their curricula, and such content is now included in 90% of medical schools in the US [5], 59% of medical schools in Britain [6] and 52% of medical schools in German-speaking countries [7]. Despite these promising numbers, S/H content is still being provided in a nonstructured, nonmandatory and fragmented way, relying mostly on theoretical classes with minimal practical exposure [5].

According to a study published in 2012 [8], the numbers were even lower in Brazil compared to the rest of the world, with approximately 40% of medical schools providing S/H content in the curriculum (4.6% with required and 5.8% with elective S/H courses). However, since that period, there have been several changes in Brazil that have prompted the incorporation of S/H content in different contexts, such as S/H guidelines from different Brazilian societies, topics addressed in medical events and increasing research and awareness by faculty and students [9–11].

Within this context, a new study is needed to understand how the context of S/H has changed in Brazil over 10 years. Exploration of the teaching methods, types of content, gaps in the delivery of such content and reasons for a medical school to offer or not offer such courses could provide valid information for medical educators and health care faculty worldwide by identifying new teaching trends and understanding how these issues could be incorporated into national and international contexts.

Specifically, in the case of Brazil, this study could serve as a starting point to understand which factors and barriers could be related to the incorporation of S/H content, highlighting possible changes in the teaching of this subject 10 years after the previous study [8] and supporting the use of these findings to promote and design a national S/H competence-based curriculum.

Therefore, in the present study, we aimed to investigate the current status of S/H teaching in all Brazilian medical schools, evaluating the extent of teaching, the type of content addressed, the strategies used, the opinions of medical directors/deans on the topic and the factors associated with the incorporation of the topic in the curriculum.

## Methods

This was a nationwide survey carried out between July and December 2021, including all medical schools in Brazil. The study strictly adhered to the STROBE checklist, as shown in the Supplementary Material.

Eligibility criteria:

Public or private medical schools that were officially registered in and accredited by the Brazilian Ministry of Education and offered medical training to students at the time of the study were included. Schools that were not registered or did not have students at the time of the study were excluded.

Participants were representatives of officially registered Brazilian medical schools (i.e., directors, coordinators/deans or medical teachers indicated by directors or coordinators) who gave consent and agreed to participate in the surveys.

Procedures:

To obtain an updated and comprehensive list of medical schools, an initial website (http://www.escolasmedicas.com.br/) was consulted in 2021. This website, although not from an official government body, probably provides the most important information available concerning medical schools in Brazil, since it is constantly updated and contains important information concerning the year of founding, names of school representatives, phone numbers and, in some cases, course descriptions. The advantage of using such a list is that it includes some medical schools that recently opened, which may not be listed on official websites.

After obtaining this initial list, all data were validated according to the official websites of the Brazilian Ministry of Education (http://emec.mec.gov.br/) and Brazilian Federal Medical Council (https://portal.cfm.org.br/busca-por-escolas-medicas/). Schools not appearing in these official databases were later checked through government directives that regulate the opening and functioning of such schools in Brazil.

This initial validation resulted in 342 medical schools in Brazil available for inclusion, which is in accordance with data obtained by a recent study [12]. The contact information of representatives was then confirmed by checking the school websites and, if not available, by phoning the medical school secretary directly.

After this initial screening, emails containing a letter of invitation explaining the objectives of the study, a Survey Monkey® link access to the online questionnaire and the approval of the Ethics Research Committee were sent to the medical school representatives. The schedule for sending these emails included the following: first contact (initial emails sent), second contact (after 45 days - emails sent again), third contact (after 90 days - the medical school was phoned to ensure that the email was correct; we spoke to a secretary or representative to make them aware of the study, explaining the objectives of the study and the importance of participating. Then, a new email was sent) and fourth contact (after 135 days –other medical teachers from the same medical school were contacted to make them aware of the study and ask them to talk to the medical school’s representative. Then, emails were sent again).

Since our aim was to obtain information on S/H teaching from all medical schools and the response rate for online surveys tends to be low, we opted to include information from a wide range of sources, including the following:

- Higher accuracy information (official information).

- Emails to representatives: in the case of duplicates (for instance, a professor and a coordinator/dean both answering the questionnaire, we considered the responses of the person in the higher hierarchical job, which, in this case, is the coordinator/dean).
- Searching for the Course-Pedagogical Project (PPC) or course syllabuses of the medical schools’ curricula: The PPC is a school management document established by Brazilian federal legislation that is mandatory for Brazilian medical schools. These documents usually contain the syllabus for all disciplines, the educational strategies used in the medical school and the objectives of the course.
- Medical schools’ websites: Websites for the institutions were consulted, and all relevant information concerning S/H content and the curriculum were retrieved.
- Brazilian Associations for S/H Academic Leagues: The websites of two associations, i.e., the Brazilian Association for the Academic Leagues and Study Groups in Spirituality and Health (AALEGREES) and Brazilian Association of Academic Leagues in Complementary and Integrative Medicine (ABLASIC), were searched, and lists of the leagues registered in their associations were found. Academic leagues are groups of medical students who aim to study a subject according to the core values of the university. These students, with a common interest, can get together, invite a supervising physician, and develop essential skills based on learning, assistance, and research [13]. Using such information, it was possible to verify which S/H leagues had been established at each medical school.

b. Lower accuracy information (information provided by other medical teachers, students or internet searches).

- Consultations with members of the S/H working groups of medical organizations and medical teachers who research and teach S/H content: Information on whether S/H content is included among the medical schools was retrieved after consulting members of the S/H working groups of medical organizations and medical teachers known for S/H research and teaching.
- Consultations with medical school students: When no representatives and no medical teachers were found, medical students were consulted concerning whether their medical school curriculum included S/H content.
- Internet searches: Internet searches were used to verify whether some content was offered in the medical schools’ curricula. Some medical schools publicized their activities and leagues on social media, on nonofficial websites and in press releases. Researchers searched Google and other search engines for S/H information using terms such as “spirituality”, “religion”, “medical students”, “medical education”, “disciplines”, “course” and the name of the medical school.

Instruments:

The instrument was an online (Survey Monkey® form), self-administered questionnaire that took an average of 15 min to complete. The questionnaire was developed based on previous studies concerning the status of S/H teaching in medical schools [5, 6, 8] and included the following:

[1] Medical school characteristics, such as the name, founding year, the number of students who graduate per year, whether it is a public or private medical school and the Brazilian federative union (state).

[2] Data about the person responsible for answering the survey, including their name, position, phone number, and email.

[3] Types of S/H content available at the medical school: Representatives were asked whether there were required or elective courses addressing only S/H, other courses that address S/H in their syllabuses (e.g., an ethics course introducing a class on Jehovah’s witnesses and blood transfusion or a clinical skills course regarding how to collect a spiritual history), S/H academic leagues, other academic leagues that address S/H (example: a palliative care league), extension programs on S/H, study groups on S/H, and future planned courses on S/H not yet in the curriculum. For these activities, the following information was collected: the name of the course/activity, whether the activity was required or elective, methodological aspects, the duration of the activity, objectives, the name of the responsible professor, undergraduate year, whether the course addressed S/H issues in clinical practice or simulations, assessment methods and course/discipline syllabuses. In the case of S/H events, participants were asked whether these events were conferences, symposiums or seminars and, if available, to attach their programs.

[4] Medical school representatives’ opinions on S/H teaching: The complete questions are included in the Results section. Questions were answered using a 5-point Likert scale (totally agree, partially agree, indifferent, partially disagree and totally disagree). The representatives were asked about their opinions of the need for S/H content in the curriculum, preparedness and training of faculty regarding S/H, guidelines of S/H, and the importance of including S/H content for medical teachers and students.

[5] Barriers to the incorporation of S/H in the curriculum: A list of barriers was provided for representatives, who could choose all that applied to that context: a lack of knowledge, a lack of training, a lack of time, no space in the curriculum, the topic not being needed in medical training, a lack of institutional support, preconceptions regarding the topic, a lack of scientific evidence and no barriers.

[6] A question about the importance of including S/H content in medical education, with the response options ranging from 0 (not important) to 10 (very important).

### Statistical analysis

Descriptive statistics are presented using the frequency, percentage, mean and standard deviation. The analyses were carried out as follows: (a) Using all sources of information (n = 342 medical schools) to evaluate the most common S/H topics offered to students and their prevalence in the curricula and (b) using the information provided by the representatives (n = 150) to evaluate the most common barriers and their opinions on S/H, since these items were available only in the representatives’ questionnaires.

For the inferential analysis, only the representatives’ information (n = 150) was considered. First, the variables of the survey were categorized as follows: (a) Items related to the course structure: founding year (years), the number of students who graduate per year (‘up to 90, inclusive’ or ’91 or more’), whether the medical school is a public or private institution (public or private) and the Brazilian administrative region (South/Southeast or Central West/Northeast/North); (b) Barriers reported by the representatives (“yes” or “no” for each of the barriers cited); and (c) Medical school representatives’ opinions regarding S/H teaching (categorized as “totally agree/partially agree” or “indifferent/partially disagree/totally disagree”).

Then, two logistic regression models were constructed to determine the factors associated with the following dependent variables: The willingness of medical schools to have S/H content in the curriculum (“yes” or “no”) and their willingness to have a required or elective course addressing only S/H content in the curriculum (“yes” or “no”). Logistic regression models were used to obtain the best explanatory variables that predicted the dependent variables. Additionally, a linear regression model was used for the variable “importance of including S/H content in medical education”, with the response options ranging from 0 (not important) to 10 (very important). Although this variable was negatively skewed in the Q-Q plot, based on central limit theory and according to previous evidence[14], sample sizes larger than 100 are robust to nonnormal distributions, such as the case for our data.

Before the regression models, chi-square (categorical) and t tests (continuous) were carried out between the independent variables and all of the questionnaire items (i.e., medical school variables, barriers according to the representatives, medical school representatives’ opinions regarding S/H teaching). Variables with a p < 0.10 in these associations were then selected to be entered in the regression models. Subsequently, final models were developed including only the statistically significant variables (p < 0.05), i.e., the best-fit models. Odds ratios and 95% confidence intervals were presented in the logistic regressions aiming to describe the predictors of the dependent variables (i.e., odds ratios greater than 1 indicated a higher probability of having S/H content, while those less than 1 indicated a lower probability of having S/H content).

All data were analyzed using the R version 4.2.2 statistical package, and a value of p < 0.05 was adopted as significant for the final regression models with a 95% confidence interval.

Ethical aspects:

The study was approved by the Ethics Research Committee of the University Hospital at the Federal University of Juiz de Fora, Brazil (number: CAAE: 30947620.8.0000.5133). All participants gave online informed consent. In the case of representatives, this online form was provided on the first page of the questionnaire. In the case of S/H working group members, medical teachers and students, online informed consent was obtained through a link provided to those answering the question regarding whether their medical school curriculum included S/H content.

## Results

Information was retrieved from all 342 (100%) Brazilian medical schools (Table 1). The different sources of information were as follows: 150 (43.9%) representatives were reached by email and completed the entire survey; 142 (41.5%) were recruited through the PPC or course syllabus of their medical school’s curriculum or official website; 11 (3.2%) were recruited from the Brazilian Associations for S/H Academic Leagues; 30 (8.8%) were S/H working group members; 8 (2.3%) were medical teachers who research and teach S/H (n = 8); and 1 (0.3%) was a fourth-year medical student from a countryside Brazilian medical school (n = 1). No new information was obtained through other internet searches (n = 0).

**Table 1:** “Spirituality and Health” (S/H) content in the curricula of Brazilian medical schools (2021) and sources of information (n = 342 medical schools)

Sources of information	n (%)
Representatives’ answers Course-Pedagogical Projects, syllabuses or official websites Brazilian Associations for S/H Academic Leagues Members of S/H working groups Medical teachers who research and teach S/H Medical students	150 (43.9%) 142 (41.5%) 11 (3.2%) 30 (8.8%) 8 (2.3%) 1 (0.3%)
Type of S/H content	n (%)
Any S/H content	224 (65.5%)
Required or elective courses addressing only S/H Required course addressing only S/H	72 (21.0%) 23 (6.7%)
Elective course addressing only S/H	49 (14.3%)
Other courses that address S/H in their syllabi	124 (36.3%)
Academic leagues on S/H	94 (27.5%)
Other academic leagues that address S/H	16 (4.7%)
Extension programs on S/H	35 (10.2%)
Future planned courses on S/H not yet in the curriculum	8 (2.3%)
Educational strategies used to teach S/H	n (%)
Practical S/H content with real patients	15 (4.3%)
Practical S/H content with standardized patients or role playing	28 (8.1%)
Longitudinal insertion of S/H content in different moments of formation (basic, clinical and clerkship)	2 (0.6%)
Number of different forms of S/H content in the curriculum	
0 1 2 3 4 5	118 (34.5%) 132 (38.6%) 64 (18.7%) 22 (6.4%) 5 (1.5%) 1 (0.3%)

The results are presented below in two separate analyses, one including all sources of information and the other including only the representatives’ answers.

### A) results for all sources of information (342 medical schools)

Table 1 also presents the prevalence of S/H content among all 342 medical schools. A total of 224 schools (65.5%) had S/H content in the curriculum, and 72 (21.0%) had required or elective courses addressing only S/H; among these, 23 (6.7%) had required courses addressing only S/H, and 49 (14.3%) had elective courses addressing only S/H. A total of 124 (36.3%) medical schools had other courses addressing S/H in their syllabi, 94 (27.5%) had academic leagues on S/H, 16 (4.7%) had other academic leagues that addressed S/H, 35 (10.2%) had extension programs, and 8 (2.3%) had required or elective courses addressing only S/H. The curricula of only 2 medical schools (0.6%) involved the longitudinal insertion of S/H content in different moments of formation (basic, clinical and clerkship); 15 schools (4.3%) had practical classes with real patients and 28 (8.1%) had practical S/H content with standardized patients or role playing. Most medical schools had only one form of S/H content in the curriculum (n = 132, 38.6%), 118 (34.5%) had no S/H content, 64 (18.7%) had 2 different forms of S/H content and 28 (8.2%) had 3 or more different forms of S/H content.

### B) results for the responses of medical school representatives (n = 150)

Concerning the representatives’ responses (n = 150), 60% were coordinators/deans, 10% were directors, 8% were deans for medical education, 10.7% were medical teachers indicated by the director/coordinator and 11.3% held other positions, e.g., vice coordinator, clerkship coordinator, pediatric course coordinator, and clinical cycle coordinator. The most prevalent region represented by these representatives was the southeast (44%), followed by the northeast (20%) and the south (18.7%). Most institutions were private (52.7%), with a median of 90.0 - ranging from 20 (minimum) to 220 (maximum) students per class. Respondents gave a median score of 10.0 - ranging from 1 (minimum) to 10 (maximum) for the importance of S/H content in medical education. This corresponds to 81 (54%) respondents grading this item as “very important − 10” (in a scale with possible answers: 0 = not important at all to 10 = very important).

The prevalence of S/H content in medical schools’ curricula was also obtained considering the answers of these 150 representatives. In this case, 122 (81.3%) medical schools had S/H content in the curriculum, 50 (33.3%) had required or elective courses addressing only S/H, 87 (58.0%) had other courses addressing S/H in their syllabi, 60 (40.0%) had academic leagues, 6 (4.0%) had other academic leagues that addressed S/H, 35 (10.2%) had extension programs, and 8 (5.3%) had required or elective courses addressing only S/H. The curricula of only 2 (1.3%) schools involved the longitudinal insertion of S/H content in different moments of formation (basic, clinical and clerkship).

Table 2 presents the medical school representatives’ opinions regarding S/H teaching (n = 150). Most respondents agreed or strongly agreed that spirituality influences patients’ health (96.7%), that this topic is considered important by their institution (74.0%), by medical teachers (58.7%) and by students (52.0%), that S/H should be inserted in different stages of the course (92.7%) and that sources of support (96.0%), guidelines (85.3%) and required competencies (80.7%) are needed. Nevertheless, they also believed that more (82.7%) and improved (82.7%) content on S/H is needed in their medical school curricula and that a lack of time (50%) hinders the incorporation of the topic. The representatives believed that their medical schools’ teachers were prepared to address S/H (60.7%), were willing to receive S/H training (67.3%), that it is important to develop assessment methods to evaluate S/H competencies (78.0%) and that financial resources would benefit the incorporation of the topic (76.0%). Approximately half of the respondents (52.0%) believed that S/H should be included in courses/disciplines that already exist and not in a specific course or discipline.

**Table 2:** Medical school representatives’ opinions regarding “Spirituality and Health” (S/H) (n = 150)

	Agree/Strongly Agree	Disagree/Strongly Disagree/Indifferent
More content on “Spirituality and Health” (S/H) is needed in the curriculum of the medical school	124 (82.7%)	26 (17.3%)
The incorporation of S/H in the curriculum of my medical school could be improved	124 (82.7%)	26 (17.3%)
My medical school has prepared teachers to deliver courses on S/H	91 (60.7%)	59 (39.3%)
The teachers at my medical school are willing to receive training or teaching material on S/H	101 (67.3%)	49 (32.7%)
Financial support or training for professors would help in the incorporation of S/H content into the curriculum	114 (76.0%)	36 (24.0%)
I believe that this issue should be included in courses/disciplines that already exist and that specific S/H courses/disciplines should not be created	78 (52.0%)	72 (48.0%)
I believe that S/H content should be incorporated in different stages of the course	139 (92.7%)	11 (7.3%)
The inclusion of S/H content is hampered by the extensive content provided by the medical curriculum	75 (50.0%)	75 (50.0%)
It is important that the incorporation of S/H content be supported by national curriculum guidelines	128 (85.3%)	22 (14.7%)
It is important to establish national minimum competency requirements in S/H training	121 (80.7%)	29 (19.3%)
It is important to develop assessment methods to evaluate medical students’ competencies in S/H	117 (78.0%)	33 (22.0%)
My medical school considers S/H content to be important	111 (74.0%)	39 (26.0%)
The students at my medical school consider S/H content to be important	78 (52.0%)	72 (48.0%)
The teachers at my medical school consider S/H content to be important	88 (58.7%)	62 (41.3%)
I believe spirituality has an influence on patients’ health	145 (96.7%)	5 (3.3%)
It would be valid to have online education materials for educators on how to teach S/H	144 (96.0%)	6 (4.0%)

The five most common barriers to the incorporation of S/H into the curriculum according to the medical school representatives (n = 150) (Table 3) were a lack of knowledge of the medical teachers/faculty (53.3%), a lack of time (43.3%), topic not being included in the teaching plan of the curriculum (28.7%), preconceptions regarding the topic (24.7%) and a lack of places for training (18.0%).

**Table 3:** Barriers to the incorporation of “Spirituality and Health” content into the curriculum according to medical school representatives (n = 150)

	Yes	No
Lack of knowledge of professors/faculty	80 (53.3%)	70 (46.7%)
Lack of places for training	27 (18.0%)	123 (82.0%)
Lack of time (extensive schedules)	65 (43.3%)	85 (56.7%)
Topic not included in the teaching plan of the curriculum	43 (28.7%)	107 (71.3%)
This topic is not needed in medical training	1 (0.7%)	149 (99.3%)
Lack of scientific basis	18 (12.0%)	132 (88.0%)
Lack of institutional support	25 (16.7%)	125 (83.3%)
Preconceptions regarding the topic	37 (24.7%)	113 (75.3%)
I do not see any barriers at all	40 (26.7%)	110 (73.3%)

Logistic regression models were then carried out to investigate which representative responses (n = 150) were associated with having more S/H content in the curriculum (Table 4):

**Table 4:** Association between representative responses and S/H content in the curriculum and the importance of S/H in medical education according to medical school representatives (n = 150)

Having any S/H content in the curriculum (Logistic Regression)			
	OR	95% CI	p **
Lack of time	0.18	0.03–0.92	**0.03**
Having prepared medical teachers	5.29	1.03–27.14	**0.04**
Having required and/or elective courses addressing only S/H in the curriculum (Logistic Regression)			
	OR	95% CI	p **
Lack of knowledge	0.35	0.14–0.84	**0.01**
Topic not included in the teaching plan of the curriculum	0.19	0.06–0.59	**0.004**
More S/H content is needed in the curriculum	0.17	0.05–0.62	**0.007**
Having prepared medical teachers	3.27	1.23–8.66	**0.01**
Aiming to have standardized national competency requirements	8.29	2.14–32.08	**0.002**
Importance of including S/H content in medical education (Linear Regression)			
	Estimate	SE*	p **
Lack of time	-0.41	0.20	**0.003**
Topic not included in the teaching plan of the curriculum	-0.69	0.22	**0.002**
Content should be included in general, nonspecific S/H courses	-0.45	0.19	**0.02**
Influence on patients’ health	2.94	0.57	**< 0.0001**
Aiming to have national S/H guidelines	0.99	0.29	**< 0.0001**
Feeling that there is a lack of institutional support	0.62	0.27	**0.02**
Feeling that others have preconceptions regarding the topic	0.48	0.23	**0.03**
Students consider it important	0.77	0.20	**0.0001**

* SE: Standard Error, ** bold indicates significance at p < 0.05

- Having any S/H content in the curriculum: Inversely associated with a “lack of time” (OR = 0.18, p = 0.03) and directly associated with “having prepared medical teachers” (OR = 5.29, p = 0.04).
- Having required and/or elective courses addressing only S/H in the curriculum: Inversely associated with a “lack of knowledge” (OR = 0.35, p = 0.01), “topic not being included in the teaching plan of the curriculum” (OR = 0.19, p = 0.004) and “more S/H content is needed in the curriculum” (OR = 0.17, p = 0.007), and directly associated with “having prepared medical teachers” (OR = 3.27, p = 0.01) and “aiming to have standardized national competency requirements” (OR = 8.29, p = 0.002).

Finally, a linear regression model was carried out (Table 4). The factors associated with a lower importance of including S/H content in medical education were a “lack of time” (Beta=-0.41, p = 0.04), the “topic not being included in the teaching plan of the curriculum” (Beta=-0.69, p = 0.002), and the “content should be included in general nonspecific S/H courses” (Beta=-0.45, p = 0.02). On the other hand, the factors associated with a greater importance of including S/H content in medical education were “believing in the influence of S/H on patients’ health” (Beta = 2.94, p = 0.001), “aiming to have national S/H guidelines” (Beta = 0.99, p = 0.001), “feeling that there is a lack of institutional support” (Beta = 0.62, 0.002), “feeling that others have preconceptions regarding the topic” (Beta = 0.48, p = 0.03) and “students considering it important” (Beta = 0.77, p = 0.001).

## Discussion

In this study, we assessed the current status of S/H teaching in Brazilian medical schools. The first finding was that the number medical schools in Brazil with S/H content in the curriculum has increased compared to that reported in a previous survey published 10 years earlier [8], in which 40% of medical schools had S/H content, far below the current number of 65.5%. Likewise, the number of required or elective courses addressing only S/H doubled from 10.4% to 2011 to 21% in 2021.

Although this comparison is valuable for supporting the increase in the number of schools including the topic in Brazil, some differences in the data collection between the studies should be highlighted. In the previous study published in 2012, 47.7% of the medical schools’ representatives answered the questionnaire, and no other sources of information were included; in the present study, 43.9% of the medical schools’ representatives answered the questionnaire and other sources of information were used to obtain information for all medical schools.

Only relying on representatives who have answered the survey may be considered a limitation, since respondents with a greater interest in the subject tend to answer the questionnaire more frequently, leading to an overestimation of the numbers. This hypothesis was supported by our findings when comparing the responses of the 150 representatives and the information obtained for all 342 medical schools (i.e., 81% versus 65.5% of medical schools with S/H content and 33.3% versus 21% with required or elective S/H courses). Aiming to minimize such problems, we decided to adopt a more conservative approach, including all sources of information, which resulted in lower S/H content percentages but, on the other hand, more representativeness.

This growing interest of Brazilian medical schools regarding the topic could be justified as follows. First, several Brazilian medical organizations, such as the Brazilian Psychiatric Association, the Brazilian Cardiology Society, the Brazilian Society of Clinical Oncology and the Brazilian Medical Education Association, have recommended the incorporation of spirituality in clinical practice and teaching [9–11]. Second, Brazil has emerged as one of the top players in S/H research worldwide [1]. Finally, medical students’ and medical residents’ interest in S/H has been increasing, and several academic “leagues” have been created by medical students to overcome the lack of formal training on this topic [15, 16].

When we compare these numbers with those other countries, the numbers are higher in Brazil than in the UK (59%)[6] and in German-speaking countries (52%)[7] but lower than in the USA (90%) [5]. Indeed, the USA is the most prolific country in the field of S/H and has achieved these numbers through a combination of factors, including the high prevalence of religious individuals, promoting research on this topic and funding by organizations to increase S/H teaching [2, 17–19].

Despite the promising numbers found in Brazil, it is important to highlight that there are several challenges and barriers to the full incorporation of S/H content in medical training, which includes the way that S/H content is taught. Although required courses addressing only S/H have the advantages of focusing on the topic and being able to provide intensive training for students [20], they have the weaknesses of being nonlongitudinal, and students are not exposed to the content in the later stages of the course [21]. These problems are even worse for elective S/H courses that tend to include only students who are more interested in the subject. In this context, other courses addressing S/H in their syllabi are interesting opportunities for continued learning, since it includes all students, and the content could be integrated into modules and disciplines throughout the curriculum. However, the success of such an approach depends on what and how often this content is offered to students. For instance, a single S/H class in the curriculum is not enough to fulfill all the competencies needed. However, courses with content integrated in different stages could fulfill these competencies.

In our survey, despite 65.5% of medical schools including S/H content, most provided content in a fragmented way and relied on single exposures, weakening students’ learning. Unfortunately, only 2 Brazilian medical schools reported longitudinal insertions in their curricula. Likewise, we found that most S/H content was theoretical with few practical interventions. These problems have also been identified in other international contexts, such as in the USA [5] and UK [6]. In Brazil, a previous study [21] showed that some medical students did not even know the meaning of the term “spiritual history” or how to address spiritual issues in clinical practice, highlighting the lack of practical classes. Future S/H discussions should draw attention to promoting S/H exposure at different moments of medical training and stimulate practical educational strategies.

Another important aspect shown in our analysis was that 27.5% of medical schools had academic leagues on S/H and 4.7% had leagues that address S/H, with this initiative being the second most common insertion of S/H in the curricula of Brazilian medical schools. When the curriculum fails to fulfill the competencies needed, academic leagues appear as opportunities to bring students closer to the topic of interest [13, 21]. This educational approach is very common in Brazil, and in the case of S/H, it was one of the most important drivers for the development of S/H teaching in medical schools, since several S/H courses or disciplines started as academic leagues [22, 23].

It is important to highlight that several barriers to S/H content incorporation were reported, such as a lack of knowledge of the medical teachers/faculty, a lack of time, and the topic not being included in the teaching plan of the curriculum. The same barriers observed in our study were also reported by physicians [16, 24], highlighting that these barriers should be considered and addressed by educators. Providing S/H training for medical teachers is one of the most important ways to reduce stigmatization and promote the incorporation of S/H content into theoretical and practical classes since training reduces the lack of knowledge and time and encourages faculty to discuss the curriculum [20, 25, 26].

Concerning the opinions of representatives on S/H issues, the study showed that most medical school representatives had a favorable view of including spirituality content in the medical curriculum. However, they agreed that their schools were not providing appropriate teaching on this topic and that their medical teachers were willing to receive training if available.

Finally, this study investigated factors related to the incorporation of S/H content into the curriculum and the importance of this topic for medical school representatives. It was found that a “lack of time”, a “lack of knowledge” and “the topic not being included in the teaching plan of the curriculum” were associated with lower incorporation of S/H content and/or lower importance given to the topic. On the other hand, “having prepared medical teachers”, students considering S/H important and “aiming to have competency requirements” were associated with greater incorporation of S/H content and/or greater importance.

“Aiming to have national competency requirements” presented the highest odds ratio in the logistic regression and should be discussed further. Competencies are important tools to increase the incorporation of S/H content into the curriculum and to standardize teaching. Therefore, it is important to provide medical schools with minimum requirement goals for their students, since the lack of standardization of S/H content in Brazil was notable in this study. Additionally, some of the most known problems of S/H teaching are the resistance of faculty and misconceptions toward the topic. Therefore, having competency requirements supported by recognized national organizations (e.g., the Brazilian Medical Education Association, the Brazilian Medical Residency Commission and the Brazilian Medical Council) could promote the incorporation of the topic in medical schools since external regulations are important ways to guarantee that such a topic is available for students. In this context, previous initiatives, such as those carried out in the USA [2, 27], could also be valuable to Brazil, allowing educators to discuss the role of S/H in clinical practice and education and work together to build a competency-based curriculum. This curriculum could be developed through a broad discussion with the scientific and educational community, aiming to include the cultural, religious and spiritual aspects of the Brazilian population, providing an integrative and sensitive approach to health care.

Another important factor associated with S/H content in our survey was “having prepared medical teachers”. In our view, this is not surprising since schools with S/H content tend to have medical teachers who have an interest in and are teaching S/H. Nevertheless, these results also highlight the pivotal role of S/H training for medical school faculty, which should be available for all medical teachers and not only for those teaching specific S/H courses. Spiritual and religious issues could appear at different stages of medical training (e.g., religious ethical issues in surgical inpatients, spiritual beliefs in geriatric outpatients and religious struggles in mothers grieving the loss of a pregnancy), and medical teachers should be able to address these ethical and religious dilemmas in medical classes and practical encounters. This could be achieved by formal training but also by stimulating the spiritual approach in the clinical practice of these professionals through extension projects or by including the spiritual dimension in service certifications when evaluating clinical care quality.

Finally, linear regression showed the feeling that there is a “lack of institutional support” and that “others have preconceptions regarding the topic” were associated with greater importance of S/H in medical education. Although this view seems contradictory at first, due to the cross-sectional nature of the present survey, our hypothesis is that respondents who confer greater importance of S/H content tend to be those who have more familiarity and have addressed this issue more in clinical practice and medical education [21]. Thus, these participants may feel more frustrated with the lack of support from the institution and feel that their peers have preconceptions.

Some limitations should be considered when interpreting our findings. First, the information was retrieved in many ways, with different levels of accuracy. The opinions of representatives, PPCs and official websites tend to be more accurate since these are official information sources. On the other hand, information obtained from a single professor or student lacks accuracy since they may not have a complete understanding of the curriculum. Therefore, we believe that numbers could be even higher if more accurate information was retrieved. Second, although PPCs are official and trustful documents, there are some delays in updating information for these documents. Likewise, some private institutions may not publicize these documents. Finally, the opinions provided by representatives may not reflect the opinions of other medical teachers and students from the institution.

This study also has several educational implications. Our study revealed that there is a lack of longitudinal S/H teaching in the curricula of Brazilian medical schools. Thus, medical educators should prioritize such teaching for their students, aiming to provide content to all students, not only those interested in this topic. Likewise, the lack of practical activities (simulations and real patients) was an important problem shown in this study. Educators should promote activities, such as history-taking and S/H communication skills, to make students competent in addressing such topics in clinical practice and avoiding common barriers, such as fear of offending patients and fear of imposing their own beliefs [15, 20]. Furthermore, the dissemination of S/H activities in medical congresses, guidelines and financial support for S/H teaching by nonprofit organizations may help in the future incorporation of this topic in Brazil and all over the world.

## Conclusion

The present study revealed an increase in the S/H content in Brazilian medical schools. Most medical school representatives agreed that this issue is important for medical training and is needed in the curriculum. However, there are also several barriers to its implementation, such as a lack of knowledge of medical teachers/faculty, a lack of time, and the topic not being included in the teaching plan of the curriculum. The most important factors that influence the incorporation of S/H content into the curriculum of medical schools were representatives’ opinions, a lack of time and knowledge, professor preparedness and standardized national competency requirements. These results could help medical educators promote S/H educational strategies and the incorporation of S/H content into curricula worldwide.

## Electronic supplementary material

Below is the link to the electronic supplementary material.

STROBE Statement?checklist of items that should be included in reports of observational studies

## Data Availability

The datasets used and/or analyzed during the current study are available from the corresponding author on reasonable request.

## References

[1] Lucchetti G, Lucchetti AL (2014). Spirituality, religion, and health: over the last 15 years of field research (1999–2013). Int J Psychiatry Med.

[2] Puchalski CM, Blatt B, Kogan M, Butler A (2014). Spirituality and health: the development of a field. Acad Med.

[3] Sajja A, Puchalski C (2018). Training Physicians as Healers. AMA J Ethics.

[4] Balboni MJ, Bandini J, Mitchell C (2015). Religion, spirituality, and the hidden curriculum: Medical Student and Faculty reflections. J Pain Symptom Manage.

[5] Koenig HG, Hooten EG, Lindsay-Calkins E, Meador KG (2010). Spirituality in medical school curricula: findings from a national survey. Int J Psychiatry Med.

[6] Neely D, Minford EJ (2008). Current status of teaching on spirituality in UK medical schools. Med Educ.

[7] Taverna M, Berberat PO, Sattel H, Frick E (2019). A Survey on the integration of spiritual care in medical schools from the german-speaking faculties. Adv Med Educ Pract.

[8] Lucchetti G, Lucchetti AL, Espinha DC (2012). Spirituality and health in the curricula of medical schools in Brazil. BMC Med Educ.

[9] Toloi DdA, Landeiro LCG, Gadia R (2022). Spirituality in oncology-a consensus by the brazilian society of clinical oncology. Brazilian J Oncol.

[10] Precoma DB, Oliveira GMMd, Simao AF (2019). Updated cardiovascular prevention guideline of the brazilian society of Cardiology-2019. Arquivos brasileiros de cardiologia.

[11] Almeida AM, Sharma A, Van Rensburg BJ, Verhagen PJ, Christopher CC (2018). Posicionamento da Associação Mundial de Psiquiatria sobre espiritualidade e religiosidade em psiquiatria. Debates em Psiquiatria.

[12] Pereira DVR, Fernandes DdLR, Mari JF, Lage ALdF, Fernandes APPC (2021). Mapping of medical schools: the distribution of undergraduate courses and annual vacancies in brazilian cities in 2020. Revista Brasileira de Educação Médica.

[13] Matheus BT. The important role of academic leagues (extensions) in Brazilian medical education. In:SciELO Brasil, 2019:98–99. 10.1590/1806-9282.65.2.98 30892427

[14] Lumley T, Diehr P, Emerson S, Chen L (2002). The importance of the normality assumption in large public health data sets. Annu Rev Public Health.

[15] Lucchetti G, de Oliveira LR, Koenig HG, Leite JR, Lucchetti AL (2013). Medical students, spirituality and religiosity–results from the multicenter study SBRAME. BMC Med Educ.

[16] Vasconcelos A, Lucchetti ALG, Cavalcanti APR (2020). Religiosity and spirituality of Resident Physicians and Implications for clinical practice-the SBRAMER Multicenter Study. J Gen Intern Med.

[17] Lucchetti G, Lucchetti ALG, Puchalski CM (2012). Spirituality in Medical Education: global reality?. J Relig Health.

[18] Park CL, George JR, Awao S (2022). US Federal Investment in Religiousness/Spirituality and Health Research: a systematic review. Religions.

[19] Demir E (2019). The evolution of spirituality, Religion and Health Publications: yesterday, today and tomorrow. J Relig Health.

[20] Osório IHS, Gonçalves LM, Pozzobon PM (2017). Effect of an educational intervention in “spirituality and health” on knowledge, attitudes, and skills of students in health-related areas: a controlled randomized trial. Med Teach.

[21] Esperandio MRG, de Souza YQ, Nadalin O, Hefti R (2021). Spirituality in clinical practice: the perspective of brazilian medical students. J Relig Health.

[22] Costa MS, Dantas RT, Alves CGdS, Ferreira ER (2019). Silva AFd. Spirituality and religiosity: knowledge of medical students. Revista Bioética.

[23] Damiano RF, Lucchetti ALG, Lucchetti G. Teaching “spirituality and health” in medical and healthcare undergraduate courses. HU rev 2018:515–525.

[24] Ellis MR, Vinsor D, Ewigman B (1999). Addressing spiritual concerns of patients: family physicians’ attitudes and practices. J Fam Pract.

[25] Hiatt MA, Reber JS, Wilkins AL, Ferrell J (2021). Incorporating spirituality in the Classroom: Effects on Teaching Quality Perception. Int J Innovative Teach Learn High Educ (IJITLHE).

[26] Best MC, Jones K, Washington J et al. Evaluation of the Interprofessional Spiritual Care Education Curriculum in Australia: Online. Palliat Support Care 2022:1–9. 10.1017/S1478951522000244 35301965

[27] Puchalski CM, Vitillo R, Hull SK, Reller N (2014). Improving the spiritual dimension of whole person care: reaching national and international consensus. J Palliat Med.

