# Supplementary material for: The current status of “spirituality and health” teaching in Brazilian medical schools: a nationwide survey
Source: BMC Med Educ. 2023 Mar 20;23:172. doi: 10.1186/s12909-023-04153-z (PMC10029158; doi:10.1186/s12909-023-04153-z)
Supplement: Supplementary file 1 — STROBE Statement?checklist of items that should be included in reports of observational studies [file 12909_2023_4153_MOESM1_ESM.docx]

STROBE Statement—checklist of items that should be included in reports of observational studies

|  | Item No. | | Recommendation | | | Pages |  |
| --- | --- | --- | --- | --- | --- | --- | --- |
| **Title and abstract** | 1 | | (*a*) Indicate the study’s design with a commonly used term in the title or the abstract | | | 2 |  |
|  |  |  | (*b*) Provide in the abstract an informative and balanced summary of what was done and what was found | | | 2 |  |
| **Introduction** |  | |  | | |  |  |
| Background/rationale | 2 | | Explain the scientific background and rationale for the investigation being reported | | | 3-4 |  |
| Objectives | 3 | | State specific objectives, including any prespecified hypotheses | | | 4 |  |
| **Methods** |  | |  | | |  |  |
| Study design | 4 | | Present key elements of study design early in the paper | | | 4 |  |
| Setting | 5 | | Describe the setting, locations, and relevant dates, including periods of recruitment, exposure, follow-up, and data collection | | | 4,6-8 |  |
| Participants | 6 | | (*a*) *Cross-sectional study*—Give the eligibility criteria, and the sources and methods of selection of participants | | | 4-5, 7-8 |  |
|  |  |  | (*b*) *Cohort study*—For matched studies, give matching criteria and number of exposed and unexposed  *Case-control study*—For matched studies, give matching criteria and the number of controls per case | | | N/A |  |
| Variables | 7 | | Clearly define all outcomes, exposures, predictors, potential confounders, and effect modifiers. Give diagnostic criteria, if applicable | | | 8-9, 10-11 |  |
| Data sources/ measurement | 8* | | For each variable of interest, give sources of data and details of methods of assessment (measurement). Describe comparability of assessment methods if there is more than one group | | | 8-9, 10-11 |  |
| Bias | 9 | | Describe any efforts to address potential sources of bias | | | 11 |  |
| Study size | 10 | | Explain how the study size was arrived at  *# All medical schools (entire population) was included* | | | N/A# |  |
| Quantitative variables | 11 | | | Explain how quantitative variables were handled in the analyses. If applicable, describe which groupings were chosen and why | | 10-11 |  |
| Statistical methods | 12 | | | (*a*) Describe all statistical methods, including those used to control for confounding | | 10-11 |  |
|  |  |  |  | (*b*) Describe any methods used to examine subgroups and interactions | | N/A |  |
|  |  |  |  | (*c*) Explain how missing data were addressed | | N/A |  |
|  |  |  |  | (*d*) *Cross-sectional study*—If applicable, describe analytical methods taking account of sampling strategy | | N/A |  |
|  |  |  |  | (*e*) Describe any sensitivity analyses | | N/A |  |
| **Results** |  | | |  | |  |  |
| Participants | 13* | | | (a) Report numbers of individuals at each stage of study—eg numbers potentially eligible, examined for eligibility, confirmed eligible, included in the study, completing follow-up, and analysed | | 12-14 |  |
|  |  |  |  | (b) Give reasons for non-participation at each stage | | 12 |  |
|  |  |  |  | (c) Consider use of a flow diagram  *#Described in the text* | | N/A# |  |
| Descriptive data | 14* | | | (a) Give characteristics of study participants (eg demographic, clinical, social) and information on exposures and potential confounders | | 13 |  |
|  |  |  |  | (b) Indicate number of participants with missing data for each variable of interest | | 12 |  |
|  |  |  |  | (c) *Cohort study*—Summarise follow-up time (eg, average and total amount) | | N/A |  |
| Outcome data | 15* | | | *Cohort study*—Report numbers of outcome events or summary measures over time | | N/A |  |
|  |  |  |  | *Case-control study—*Report numbers in each exposure category, or summary measures of exposure | | N/A |  |
|  |  |  |  | *Cross-sectional study—*Report numbers of outcome events or summary measures | | 11-14 and Tables 1-3 |  |
| Main results | 16 | | | (*a*) Give unadjusted estimates and, if applicable, confounder-adjusted estimates and their precision (eg, 95% confidence interval). Make clear which confounders were adjusted for and why they were included | | 15-16 and Table 4 |  |
|  |  |  |  | (*b*) Report category boundaries when continuous variables were categorized | | 10-11 |  |
|  |  |  |  | (*c*) If relevant, consider translating estimates of relative risk into absolute risk for a meaningful time period | | N/A |  |
| Other analyses | | 17 | | | Report other analyses done—eg analyses of subgroups and interactions, and sensitivity analyses | N/A | |
| **Discussion** | |  | | |  |  | |
| Key results | | 18 | | | Summarise key results with reference to study objectives | 16-22 | |
| Limitations | | 19 | | | Discuss limitations of the study, taking into account sources of potential bias or imprecision. Discuss both direction and magnitude of any potential bias | 21-22 | |
| Interpretation | | 20 | | | Give a cautious overall interpretation of results considering objectives, limitations, multiplicity of analyses, results from similar studies, and other relevant evidence | 22-23 | |
| Generalisability | | 21 | | | Discuss the generalisability (external validity) of the study results | 22 | |
| Other information | | | | |  |  |  |
| Funding | | 22 | | | Give the source of funding and the role of the funders for the present study and, if applicable, for the original study on which the present article is based | 23 | |
